# Measurement of Organic Chemical Refractive Indexes Using an Optical Time-Domain Reflectometer

**DOI:** 10.3390/s120100481

**Published:** 2012-01-05

**Authors:** Chien-Hung Yeh, Chi-Wai Chow, Jiun-Yu Sung, Ping-Chun Wu, Wha-Tzong Whang, Fan-Gang Tseng

**Affiliations:** 1 Information and Communications Research Laboratories (ICL), Industrial Technology Research Institute (ITRI), Hsinchu 31040, Taiwan; 2 Department of Photonics and Institute of Electro-Optical Engineering, National Chiao Tung University, Hsinchu 30010, Taiwan; E-Mails: cwchow@faculty.nctu.edu.tw (C.-W.C.); tom7721.eo96@g2.nctu.edu.tw (J.-Y.S.); depew007@yahoo.com.tw (P.-C.W.); 3 Department of Materials Science and Engineering, National Chiao Tung University, Hsinchu 30010, Taiwan; E-Mail: wtwhang@cc.nctu.edu.tw; 4 Department of Engineering and System Science, National Tsing Hua University, Hsinchu 30013, Taiwan; E-Mail: fangang@ess.nthu.edu.tw

**Keywords:** fiber sensor, OTDR, refractive index (RI)

## Abstract

In this investigation, we propose and experimentally demonstrate a method for measuring the refractive index (RI) of liquid organic chemicals. The scheme is based on a single-mode fiber (SMF) sensor and an optical time-domain reflectometer (OTDR). Here, due to the different reflectance (R) between the SMF and organic liquid chemicals, the reflected power level of the backscattering light (BSL) measured by the OTDR would be different. Therefore, we can measure the RI of chemical under test via the measured BSL level. The proposed RI sensor is simple and easy to manipulate, with stable detected signals, and has the potential to be a valuable tool for use in biological and chemical applications.

## 1. Introduction

Refractive index (RI) is one of the important optical parameters of materials. RI sensors have attracted considerable attention in biological and chemical applications. This is because other parameters such as the density, concentration, temperature, and stress can be sensed and detected by measuring the RI [1]. Recently, many optical sensors for RI detection have been proposed, including fiber grating sensors [2–4], fiber surface plasmon resonance (SPR) sensors [5], photonics crystal waveguide sensors [6], microfibers-based sensors [7], and partially stripped cladding fiber sensor [8] *etc*. Fiber-based optical sensors are promising due to their inherent immunity to electromagnetic interference, safety in hazardous or explosive environments, high sensitivity and the possibility of enabling long distance measurements [9,10].

In this demonstration, we propose and demonstrate a RI measurement method using an optical time-domain reflectometer (OTDR)-based fiber sensor. The proposed measuring scheme can detect the RI of different liquid organic chemicals according to the measured reflected power level of the backscattered light (BSL) from an OTDR via a single-mode fiber (SMF)-based sensor. Based on the observed power level of BSL, we can easily obtain the RI of different organic chemicals. The reflected optical signal is measured using a commercially available OTDR; hence expensive lock-in-amplifiers as reported in [8] are not required. The sensor head is a standard SMF and is easily replaceable; hence special and tailor-made sensors, such as using photonics crystal [6] or microfibers [7] are not required. The proposed scheme is simple and provides quick measurement results.

## 2. Principles

An OTDR is used to measure the properties of an optical fiber. An optical pulse is first generated in the OTDR and is coupled into the fiber. Then the backscattered and reflected optical power is received by a photodiode at the input end as [11]:

(1) $$P_{\mathrm{backscattering}}\;(x)=\frac{1}{2}\;v\;\cdot \;s\;\cdot \;f\;\cdot \;c\;\cdot \;\tau \;\cdot \;P(0)\cdot \;e^{-2\alpha x}$$

where *x* is the position relative to input end in the fiber, *v* the group velocity of the pulse, *s* the light-scattering coefficient, *f* the fraction of scattered light captured at the input end, *c* the coupling efficiency, *τ* the pulse width, *α* the attenuation coefficient.

While the output end of the fiber is immersed into an unknown chemical, the optical pulse is reflected at the interface between media of different RIs. Based on Fresnel equation, the reflectance of light can be expressed as:

(2) $$R={\left(\frac{n_{\mathrm{eff}}-n_{\mathrm{material}}}{n_{\mathrm{eff}}+n_{\mathrm{material}}}\right)}^{2}$$

where *n_eff_* is the effective RI of the fiber, *n_material_* the RI of the measured material. Combining Equations (1) and (2), we approximate the reflection power received by PD as:

(3) $$P_{\mathrm{reflected}}\;(L)=R\;\cdot \;P(0)\;\cdot {\left(1-\frac{1}{2}v\;\cdot \;s\;\cdot \;f\;\cdot \;c\;\cdot \;\tau \;\cdot \;e^{-2\alpha L}\right)}^{2}$$

where *L* is the total length of the fiber. From Equation (3), it is clear that the reflected power is promotional to reflectance. Thus, we can conclude that, at point where reflection occurs, the OTDR received power level is:

(4) $$P_{\mathrm{OTDR}}\;(L)\propto R={\left(\frac{n_{\mathrm{eff}}-n_{\mathrm{material}}}{n_{\mathrm{eff}}+n_{\mathrm{material}}}\right)}^{2}$$

**Figure 1.**
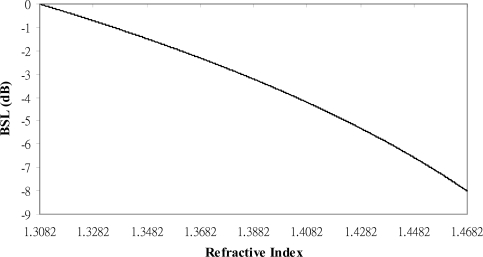
Simulation for the normalized OTDR measured power level *versus* material RI.

From Equation (4), we can relate the OTDR measured power level with the material RI we are interested in. Figure 1 shows the curve for the normalized OTDR measured power level *versus* material RI.

## 3. Experiments and Discussion

**Figure 2.**
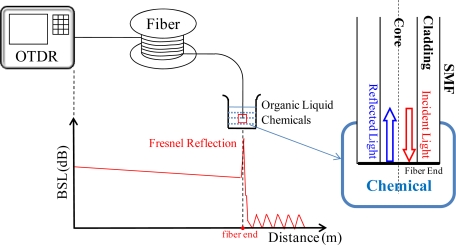
Experimental setup for RI measurement of organic liquid chemical by using OTDR-based fiber sensor head. The below schematic is the OTRD trace. The terminal facet of SMF is employed to serve as the sensing head and inserts in organic liquid chemical for RI detection, as shown in insert.

Figure 2 shows the proposed experimental setup for detecting the RI of liquid organic chemicals. For general use in optical communication network, an optical pulse from the OTDR is launched into the optical fiber. By measuring the backscattered light (BSL), the OTDR can locate fiber breaks or splice losses [12]. In the experiment, the OTDR is used to connect to a 1,100 m long single mode fiber (SMF) for RI detection. In the measurement, the sensor head is a standard SMF (attenuation 0.2 dB/km). The terminal facet of SMF is employed to serve as the sensor head and immersed into the organic liquid chemicals for RI detection, as shown in inset of Figure 2. The sensor head should be flat and is prepared by a typical fiber cleaver. In this experiment, the cladding and core diameters of SMF are 125 μm and 8.2 μm, respectively. The effective index (*n_eff_*) of the SMF is nearly 1.4682. Here, eight liquid chemicals of known RI were used, namely water, ethyl acetate, *n*-hexane, methyl ethyl ketone, heptane, THF, cyclohexanone and ethanolamine. Their corresponding RIs are 1.3333, 1.3727, 1.3750, 1.3800, 1.3870, 1.4070, 1.4500 and 1.4540, respectively. According to the Fresnel reflection equation, the reflected optical power depends on the RIs between two media. When the correlation between the reflected BSL optical powers of several chemical samples and their corresponding RIs is built, the chemical of unknown RI can be easily obtained by measuring the reflected BSL using an OTDR.

**Figure 3.**
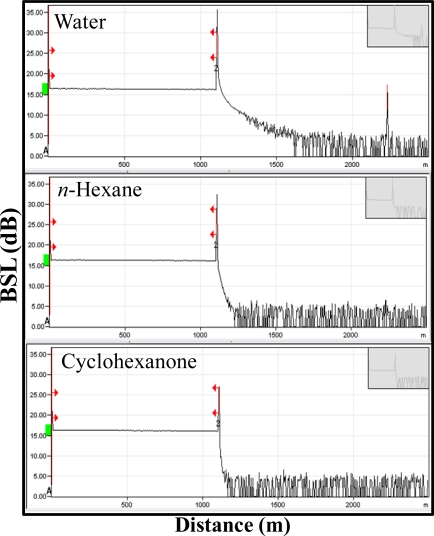
The output OTDR traces when the measured samples are water, *n*-hexane and cyclohexanone, respectively, at 1,100 m long SMF.

Figure 3 presents the output OTDR traces for three tested samples: water (RI = 1.3333), *n*-hexane (RI = 1.3750) and cyclohexanone (RI = 1.4500). We can observe from Figure 3 that when the RI of chemical sample under test is approaching the *n_eff_* of SMF, the Fresnel reflection level of BSL (the reflected optical power depends on the RIs between two media) will decrease. The wavelength and the pulse width of the launching optical pulse laser are 1,550 nm and 10 ns respectively. It is also worth mentioning that the experiment is performed using a 1,100 m SMF. This means that the proposed scheme can enable long distance and remote sensing. In this case, the OTDR is located at the central office (CO), while the sensor (SMF fiber facet) is in a remote location.

**Figure 4.**
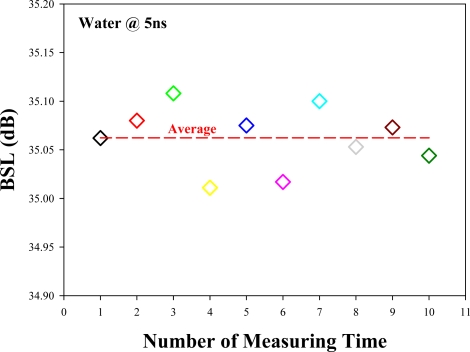
Output BLS levels from the OTDR traces at 1,100 m SMF long under 10 time measurements when the water is used for sensing and the 1,550 nm pulse laser is setup at 5 ns (red dashed line is the average value of measured BLSs).

The BSL intensity depends on the intensity of the laser pulse. Hence, in order to reduce the effect of laser power fluctuation, an average value (10 measurements of each sample) of each sample is reported in the paper. For example, we measure the BSL level of water via the proposed OTDR-based fiber sensor under 10 time measurements using a 1,550 nm pulse laser with 5 ns pulse-width, as shown in Figure 4. And the average value of the 10 time measurements is about 35.0623 dB, as also illustrated in red dash line of Figure 4.

**Figure 5.**
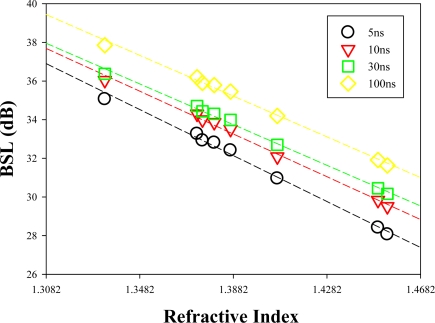
BLS levels from the OTDR traces under various testing liquid chemicals at 1,100 m SMF long, when the 1,550 nm pulse laser is setup at 5, 10, 30 and 100 ns, respectively.

As mentioned before, when the correlation between the reflected BSL optical powers of several chemical samples and their corresponding RIs is built (as shown in Figure 5), a chemical of unknown RI can be easily identified by measuring the reflected BSL using an OTDR. Here, we also characterize using different optical pulse-widths of 5, 10, 30, and 100 ns for the sensing using 1,550 nm optical pulses. In this measurement, each liquid chemical is measured via the proposed OTDR-based sensor 10 times. We can observe from Figure 5 that when the optical pulse-width increases, the measured reflected level of BSL also increases. The physical concept is that higher pulse-width will provide higher total optical power, hence higher reflected BSL level can be observed in the OTDR. We can also observe from Figure 5 that the trends of the four curves are nearly the same when using optical pulse-widths of 5, 10, 30 and 100 ns respectively. It is clear that the experimental curves and the simulation curve are similar in trend.

In this experiment, the minimum RI difference of the eight chemical samples is 0.0023 (ethyl acetate = 1.3727 and *n*-hexane = 1.3750). The corresponding Fresnel reflection differences are obtained at 0.342, 0.306, 0.278 and 0.271 dB, under the pulse widths of 5, 10, 30 and 100 ns, respectively. Hence, we can observe that shorter pulse-width would result in the better measured resolution for sensing. For a specific RI is estimated by the measured OTDR power level, the power fluctuation will make the deduced RI fluctuate. As we have shown, among all of our ten measurement values for the case of water (in Figure 4), the mean BSL is 35.0623 dB, while maximum and minimum BSLs are measured at 35.108 and 35.011 dB. If we are using linear interpolation for the estimated curve, the RI fluctuation induced by the power fluctuation is (P_fluctuation_/ΔP) × Δn. Where ΔP is the power level difference between two pre-known values; Δn is the RI difference between the corresponding points. P_fluctuation_ is the OTDR power level fluctuation. Thus, in our case, the fluctuation of our estimated RI is 6.5234 × 10^−4^. If we take the fluctuation value in the all curve is limited to this value, this will decrease our accuracy to 0.0023 + 6.5234 × 10^−4^, which is approximately to 0.003. Besides, the OTDR only gives accuracy of the second digit after the decimal point (the third digit after the decimal points is for estimation), the accuracy limit of the OTDR is 0.01. Hence we can estimate that the measurement sensitivity can be down to 2 digits after the decimal point. Furthermore, the standard deviation of the measurement is about 0.03033. By considering the laser power fluctuation and the OTDR only gives accuracy of the second digit after the decimal point. We can also estimate that the measurement sensitivity can be down to 2 digits after the decimal point.

**Figure 6.**
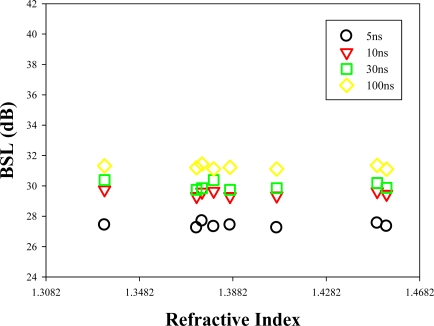
Output BLS levels from the OTDR traces under various testing liquid chemicals when the fiber sensor head with irregular facets.

The proposed OTDR-based fiber sensor head is low-cost (since it is a conventional SMF), robust and re-usable. We can rinse it with distilled water without causing any damage after finishing all the experiments. In this part, we would like to show the importance of using a fiber cleaver to prepare the sensor head to obtain useful measurement results. Figure 6 shows the measured BSL using a non-flattened (damaged) fiber sensor head, which was prepared by cutting with a pair of scissors. According the measured results in Figure 6, when the terminal facet of fiber sensor head has a non-flattened facet, we cannot measure the RI of the chemical sample. Therefore, the sensor head must be flattened for chemical sensing.

## 4. Conclusions

In this work we have proposed and experimentally demonstrated a method for measuring the RI of liquid chemicals. The scheme is based on a SMF sensor and a commercially available OTDR. The proposed RI sensor is simple and easy to manipulate. In the characterization, water and seven liquid organic chemicals were tested: water, ethyl acetate, *n*-hexane, methyl ethyl ketone, heptane, THF, cyclohexanone and ethanolamine. Their RIs are 1.3333, 1.3727, 1.3750, 1.3800, 1.3870, 1.4070, 1.4500 and 1.4540, respectively. It is also worth to mention that the experiment is performed using a 1,100 m SMF. This means that the proposed scheme can enable long distance and remote sensing. Besides, the fiber sensor head is low-cost (since it is a conventional SMF), robust and re-usable. We can rinse it with distilled water without causing any damage after finishing all the experiments.
